# Hearing loss in mice with disruption of auditory epithelial patterning in the cochlea

**DOI:** 10.3389/fcell.2022.1073830

**Published:** 2022-12-08

**Authors:** Sayaka Katsunuma, Hideru Togashi, Shuhei Kuno, Takeshi Fujita, Ken-Ichi Nibu

**Affiliations:** ^1^ Department of Otolaryngology, Hyogo Prefectural Kobe Children’s Hospital, Kobe, Japan; ^2^ Department of Biochemistry and Molecular Biology, Division of Molecular and Cellular Biology, Kobe University Graduate School of Medicine, Kobe, Japan; ^3^ Department of Otolaryngology-Head and Neck Surgery, Kobe University Graduate School of Medicine, Kobe, Japan; ^4^ PRESTO, Japan Science and Technology Agency, Kobe, Japan

**Keywords:** cochlea, nectin, mosaic pattern, auditory function, apoptosis, tight junction

## Abstract

In the cochlear auditory epithelia, sensory hair and supporting cells are arranged in a checkerboard-like mosaic pattern, which is conserved across a wide range of species. The cell adhesion molecules nectin-1 and nectin-3 are required for this pattern formation. The checkerboard-like pattern is thought to be necessary for auditory function, but has never been examined. Here, we showed the significance of checkerboard-like cellular pattern in the survival and function of sensory hair cells in the cochlear auditory epithelia of nectin-3 knockout (KO) mice. Nectin-3 KO mice showed progressive hearing loss associated with degeneration of aberrantly attached hair cells *via* apoptosis. Apoptotic hair cell death was due to the disorganization of tight junctions between the hair cells. Our study revealed that the checkerboard-like cellular pattern in the auditory epithelium provides a structural basis for ensuring the survival of cochlear hair cells and hearing function.

## Introduction

The epithelia of sensory organs have unique characteristic cellular patterns. In most sensory epithelia, the same types of sensory cells are separated from one another to form alternating mosaic patterns. The auditory epithelium of the mammalian inner ear consists of mechanosensory hair and non-sensory supporting cells (Gulley and Reese, 1976). When the luminal surface of the auditory epithelium is observed from the apical side, the sensory hair cells are arranged in ordered rows (three rows of outer and one row of inner hair cells), and each hair cell is separated by supporting cells, forming an alternating mosaic in a checkerboard-like fashion. These mosaic patterns of sensory epithelia are evolutionarily conserved among a wide range of species, not only among vertebrates but also between vertebrates and invertebrates. These observations suggest that mosaic patterns play a role in physiological function (Gulley and Reese, 1976; Togashi and Katsunuma, 2017). However, the physiological significance of sensory mosaics remains unclear.

We have previously shown that nectins regulate the formation of mosaic cellular patterns in sensory epithelia (Togashi et al., 2011; Katsunuma et al., 2016). Nectins are family of immunoglobulin-like cell adhesion molecules that can engage in homophilic and heterophilic trans-interactions. However, their heterophilic trans-interactions are much stronger than their homophilic trans-interactions (Satoh-Horikawa et al., 2000; Martinez-Rico et al., 2005). When cells expressing different types of nectins were co-cultured, they were arranged in a mosaic pattern (Togashi et al., 2006; Togashi et al., 2011; Katsunuma et al., 2016). In the auditory epithelium, sensory hair cells express nectin-1, and supporting cells express nectin-3. Nectin-3 is localized along the heterotypic junctions between hair and supporting cells, and also weakly detected at the junctions between supporting cells. Molecular interactions occur between nectin-1 in hair cells and nectin-3 in supporting cells, and the biased cell–cell adhesions between nectin-1 and -3 contribute to checkerboard-like pattern formation (Togashi et al., 2011). We have also reported that the characteristic mosaic pattern of the olfactory epithelium is cooperatively regulated by the cadherin and nectin systems (Katsunuma et al., 2016). These results suggest that the differential adhesion of nectins and cadherins is likely to be used in the mosaic pattern formation of various sensory epithelia.

In nectin-1 or nectin-3 knockout (KO) mice, two or three hair cells are aberrantly attached and the checkerboard-like pattern of the auditory epithelium is disorganized from the embryonic stage. However, the numbers of differentiated hair and supporting cells were not altered on postnatal days (P) 1 and P5 in the nectin KO cochlea (Togashi et al., 2011; Fukuda et al., 2014). Aberrantly attached hair cells in nectin-1 or nectin-3 KO mice show disturbances in the orientation and morphology of the stereocilia (Fukuda et al., 2014). These observations suggest that proper checkerboard-like cellular pattern formation is involved in the morphogenesis of hair cells and may play an important role in physiological processes. Mutations in the human nectin-1 gene are responsible for cleft lip/palate ectodermal dysplasia, Margarita Island ectodermal dysplasia, and Zlotogora–Ogür syndrome, which is characterized by cleft lip/palate, syndactyly, intellectual disability, ectodermal dysplasia, and partial deafness (Bustos et al., 1991; Suzuki et al., 2000; Sozen et al., 2001). These results suggest that nectin mutations are implicated in physiological functions; however, the pathogenesis of this disease remains unclear. In the present study, we examined the auditory phenotype of nectin-3 KO mice and found that proper checkerboard cellular patterning of the auditory epithelium is required for the survival and function of cochlear hair cells.

## Results

### Auditory defects of nectin-3 KO mice

**FIGURE 1:**
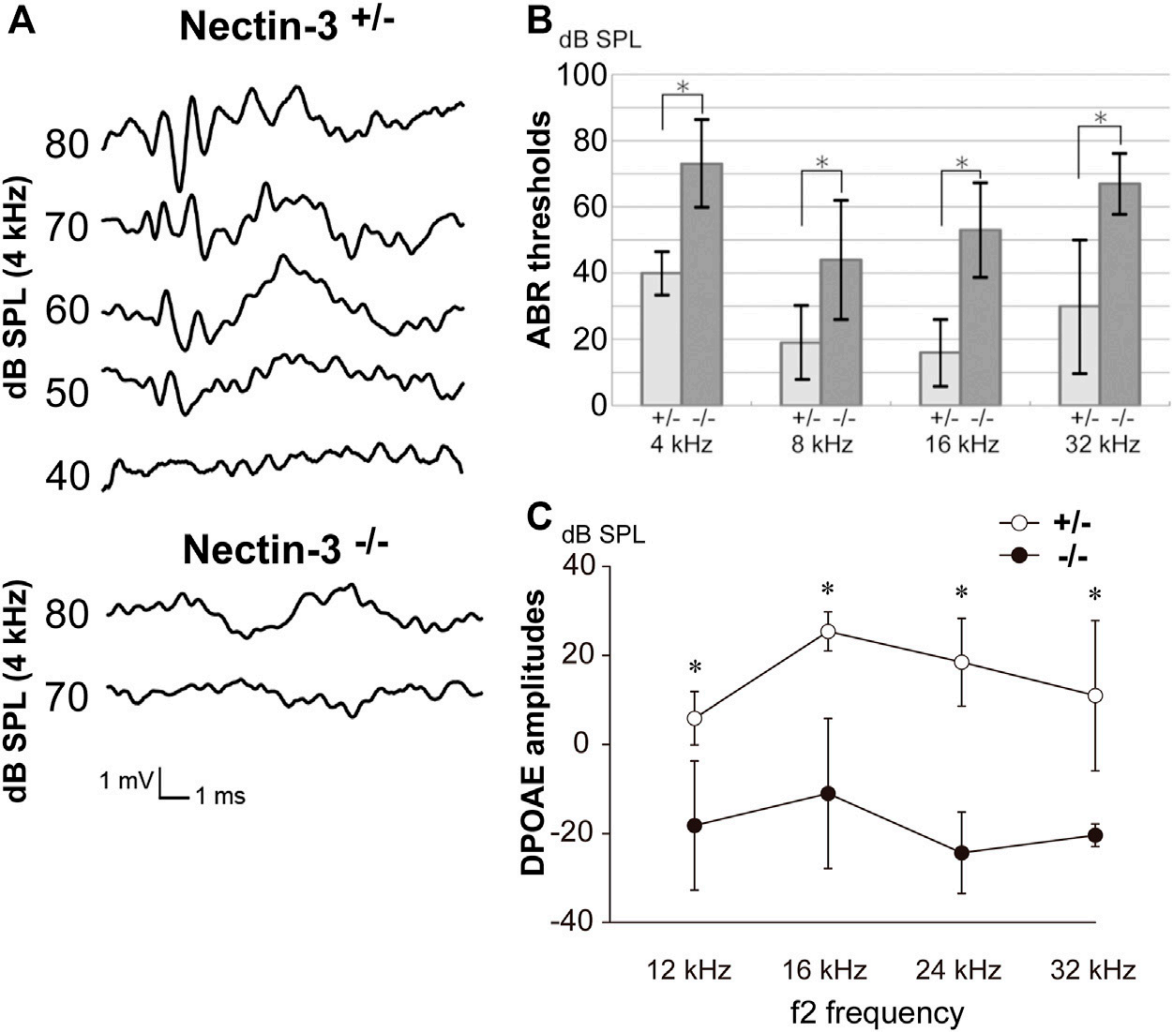
Auditory defects of nectin-3 KO mice **(A)** ABRs to stimuli of 40–80 dB SPL at 4 kHz in P28 nectin-3^+/–^ and nectin-3^−/−^ mice. Typical data for a nectin-3^+/–^ mouse and a nectin-3^−/−^ mouse are shown. **(B)** Hearing thresholds at sound frequencies of 4, 8, 16, and 32 kHz of nectin-3^+/–^ (*n* = 3) and nectin-3^−/−^ mice (n = 3) on P28. Nectin-3^−/−^ mice showed increased thresholds (55–75 dB sound pressure level (SPL)) compared with nectin-3^+/–^ mice (15–40 dB SPL). All average data on the graphs are shown as mean ± SD. In all instances, *p* values were less than 0.05 and considered to be significant. *, *p* < 0.05. **(C)** DPOAEs of P28 nectin-3^+/–^ (white dots), and P28 nectin-3^−/−^ (black dots) mice represented as a function of f2 stimulus frequencies, ranging from 12 kHz to 32 kHz. Nectin-3^−/−^ mice showed attenuated responses at this stage (mean ± SD; *n* = 8 for nectin-3^+/–^ mice, *n* = 13 for nectin-3^−/−^ mice). *, *p* < 0.05.

We examined the hearing function of nectin-3 KO mice using auditory brainstem response (ABR), a sound-induced potential in the auditory pathway from the cochlea to the brainstem. We compared ABR in nectin-3^+/–^ and nectin-3^−/−^ mice on P28. We measured the ABR to stimuli at 40–80 decibels (dB) sound pressure level (SPL) at various sound frequencies (4, 8, 16, and 32 kHz) in three sets of nectin-3^+/–^ littermates at P28. These mice showed typical ABR waveforms and normal hearing thresholds (10–40 dB SPL) (Figure 1A). We then measured the ABR to the same stimuli in three sets of nectin-3^−/−^ littermates. The ABR thresholds of nectin-3^−/−^ mice at every sound frequency (40–80 dB SPL) were significantly higher than those of nectin-3^+/–^ mice (Figures 1A,B). First wave of ABR (wave I) represents neuronal activation in cochlear spiral ganglion neurons, while second wave (wave II) and beyond represent neuronal activation in the central nervous system. Nectin-3^−/−^ mice showed no obvious wave I waveform, and the wave I amplitude was lower in nectin-3^−/−^ mice than in nectin-3^+/–^ mice (Figure 1A). These results suggested that the impaired ABR observed in nectin-3^−/−^ mice was generated in the cochlea.

To assess the ability of nectin-3^−/−^ mice to enhance cochlear sensitivity, we evaluated the distortion product otoacoustic emission (DPOAE), which represents low-level sound generated by the somatic motility of outer hair cells and emitted to the external auditory canal. The DPOAE of nectin-3^−/−^ mice was undetectable on P28 compared with that of nectin-3^+/–^ mice at all frequencies measured (12, 16, 24, and 32 kHz) (Figure 1C). These results indicated the possibility of outer hair cell dysfunction in nectin-3^−/−^ mice. Despite the apparent hearing disability, nectin-3^−/−^ mice had no detectable impairment of balance and exhibited normal gait (data not shown).

### Decreased outer hair cells in nectin-3 KO mice

**FIGURE 2:**
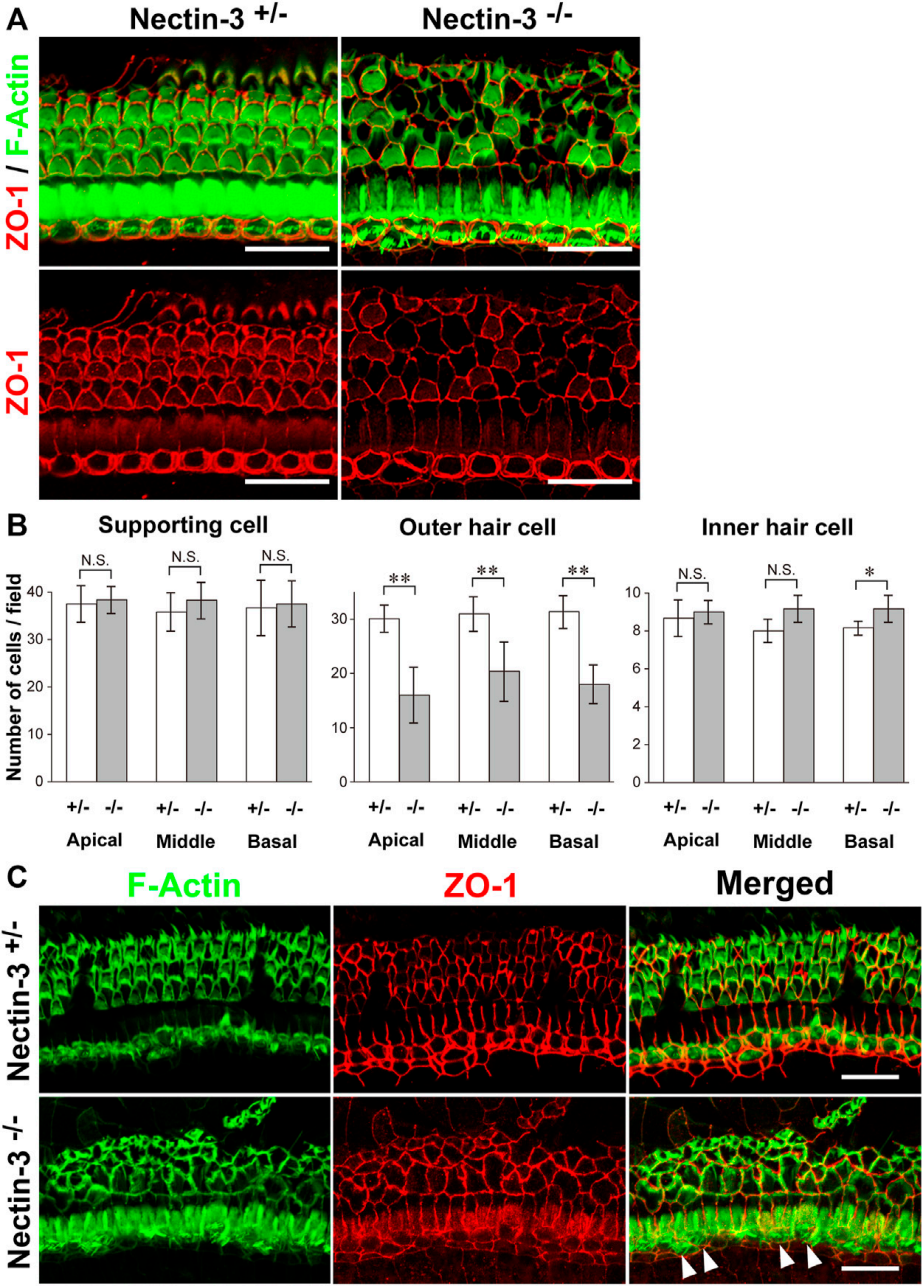
Morphology of the auditory epithelium from nectin-3 KO mice on P28 **(A)** Immunofluorescence signals for F-Actin (green) and ZO-1 (red) (upper panels) and ZO-1 (lower panels) in the middle turn of the auditory epithelium of nectin-3^+/–^ and nectin-3^−/−^ on P28. Scale bars are 25 μm. **(B)** Statistical analysis of cell number of supporting cell, outer hair cell, and inner hair cell in nectin-3^+/–^ or nectin-3^−/−^ mice on P28. (Left) Numbers of supporting cells per photographic field. (Middle) Numbers of outer hair cells per photographic field. (Right) Numbers of inner hair cells per photographic field. The results shown are the means ± SD. *, *p* < 0.05; **, *p* < 0.01, N.S., not significant. **(C)** Immunofluorescence signals for F-actin (green) and ZO-1 (red) in the middle turn of the auditory epithelium of nectin-3^+/–^ (upper panels) or and nectin-3^−/−^ (lower panels) 1 year after birth. White arrowheads indicate the inner hair cells in nectin-3^−/−^. Please note that the outer hair cells are lost in nectin-3^−/−^; however, the stereocilia of inner hair cells are still present. Scale bars are 30 μm.

To understand the mechanisms underlying hearing loss in nectin-3 KO mice, we examined the morphology and survival of hair cells in the auditory epithelium on P28. We immunostained the auditory epithelium with ZO-1 and F-actin (Figure 2A). ZO-1 was used as a marker for cell–cell junctions and F-actin for sensory hair cells. In nectin-3^+/–^ cochleae, a single row of inner hair cells and three rows of outer hair cells were regularly aligned. However, in nectin-3^−/−^ cochleae, many outer hair cells were lost, and the regular rows of outer hair cells were severely disorganized, while inner hair cells were retained. As generally observed in damaged auditory epithelium, the lost outer hair cells were replaced by supporting cells. The number of outer hair cells in nectin-3^−/−^ mice decreased to about two-thirds of that in nectin-3^+/–^ mice; however, the number of inner hair and supporting cells did not change in nectin-3^−/−^ cochlea (Figure 2B). The outer hair cells in nectin-3^−/−^ mice were reduced to the same extent from the apical to the basal turn. We also examined the degeneration of hair cells in nectin-3^−/−^ mice 1 year after birth (Figure 2C). Double-immunostaining for ZO-1 and F-actin in the auditory epithelium revealed that all outer hair cells were lost; however, most inner hair cells were retained (Figure 2C, arrowheads). These data suggest that postnatal degeneration of outer hair cells causes hearing loss in nectin-3^−/−^ mice.

### Progressive degeneration of outer hair cells in nectin-3 KO mice

**FIGURE 3:**
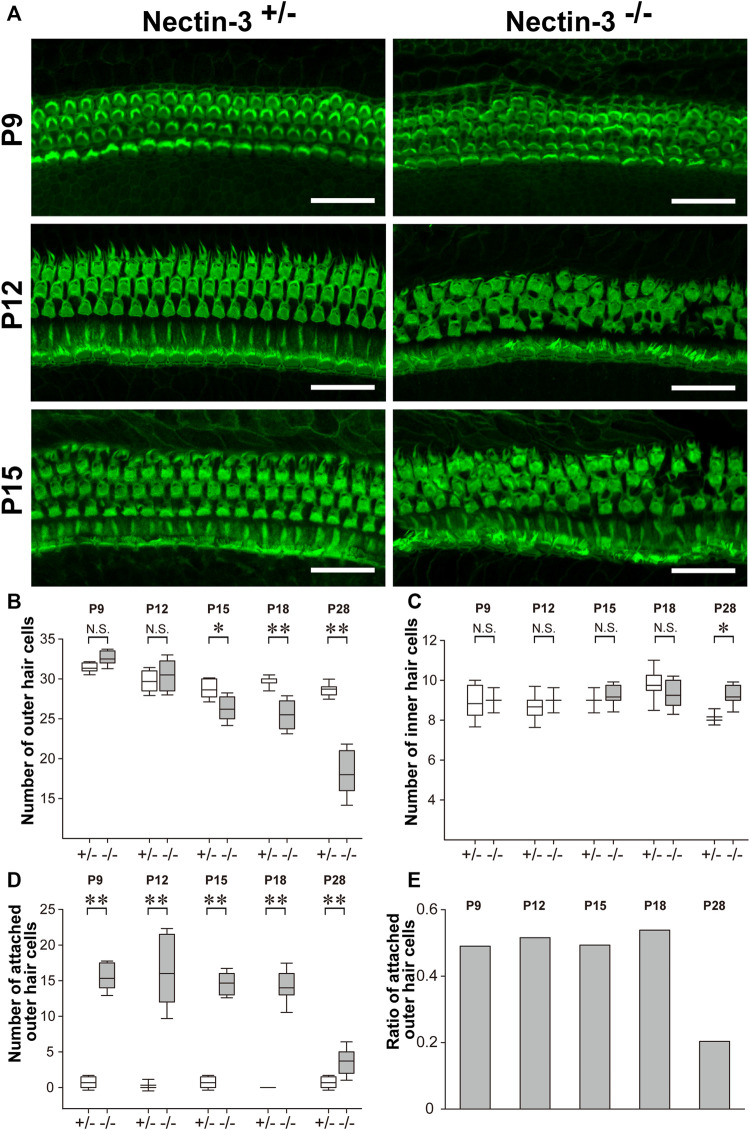
Progressive degeneration of outer hair cells in nectin-3 KO mice **(A)** Immunofluorescence signals for F-actin in the middle turn of auditory epithelium in nectin-3^+/–^ or nectin-3^−/−^ on P9, P12, and P15. Scale bars are 30 μm. **(B)** Statistical analysis of cell number change of the outer hair cell in nectin-3^+/–^ or nectin-3^−/−^ mice after birth periods, from P9 to P28. The boxes in boxplots show first and third quartiles, whiskers show 9th and 91st percentiles and the middle line of the box shows the mean. *, *p* < 0.05; **, *p* < 0.01, N.S., not significant. **(C)** Statistical analysis of cell number change of the inner hair cell in nectin-3^+/–^ or nectin-3^−/−^ mice after birth periods, from P9 to P28. The graph shows the number of attached outer hair cells in each field. **(D)** Statistical analysis of cell number change of the attached outer hair cell in nectin-3^+/–^ or nectin-3^−/−^ mice after birth periods, from P9 to P28. The graph shows the number of attached outer hair cells in each field. **(E)** The graph shows the ratio of attached outer hair cells in the total outer hair cells in each field of nectin-3^−/−^ after birth periods, from P9 to P28. Data were collected from three individuals and quantified using five fields in each individual.

We previously reported that the numbers of differentiated sensory hair and supporting cells were not altered in nectin-3^+/–^ and nectin-3^−/−^ mice on P1 and P5 (Togashi et al., 2011; Fukuda et al., 2014). We then stained the auditory epithelium derived from nectin-3^+/–^ and nectin-3^−/−^ mice with phalloidin to investigate the morphology of the auditory epithelium and counted the number of hair cells at different developmental stages (Figure 3A). On P9, in the nectin-3^−/−^ cochlea, many sensory hair cells in the third row (the outermost side) were attached to sensory hair cells of the same or different row; however, the regular rows of outer hair cells were almost maintained. From P12 to P15, many outer hair cells were attached to other sensory hair cells, and the regular rows of outer hair cells were disorganized; however, the number of sensory hair and supporting cells did not change. By P18, the surface area of the outer hair cells gradually decreased in both nectin-3^+/–^ and nectin-3^−/−^ cochleae. We also examined the morphology of the sensory hair cells at this stage. When 3D reconstitution images of the stereocilia in attached outer hair cells on P15 were observed using laser scanning microscopy, the stereocilia of unattached nectin-3^−/−^ outer hair cells were almost indistinguishable from nectin-3^+/–^, and the lengths of nectin-3^−/−^ stereocilia were not grossly affected (Supplementary Figure S1). The number of outer and inner hair and supporting cells did not differ between nectin-3^+/–^ and nectin-3^−/−^ cochlea from P9 to P15. Loss of outer hair cells was first detected on P15, and the number of outer hair cells in nectin-3^−/−^ mice significantly decreased (Figure 3B). In contrast, the number of inner hair cells in nectin-3^−/−^ mice did not change during this period (Figure 3C).

We then examined the number of attached outer hair cells in nectin-3^−/−^ mice from P9 to P28 to clarify the effect of aberrant hair cell attachment on their survival. From P9 to P18, attached outer hair cells constantly accounted for 40%–50% of the total outer hair cells (Figures 3D,E). However, attached hair cells accounted for only 20% of the total outer hair cells in nectin-3^−/−^ mice on P28. In other words, the population of attached hair cells was severely reduced between P18 and P28. In contrast, inner hair cells were retained for at least 1 year after birth. Inner hair cells of nectin-3^−/−^ mice were also aberrantly attached, although less frequently than outer hair cells (Togashi et al., 2011). These observations suggest that attached hair cells specifically decreased after P18.

### FM1-43 uptake in nectin-3 KO hair cells

**FIGURE 4:**
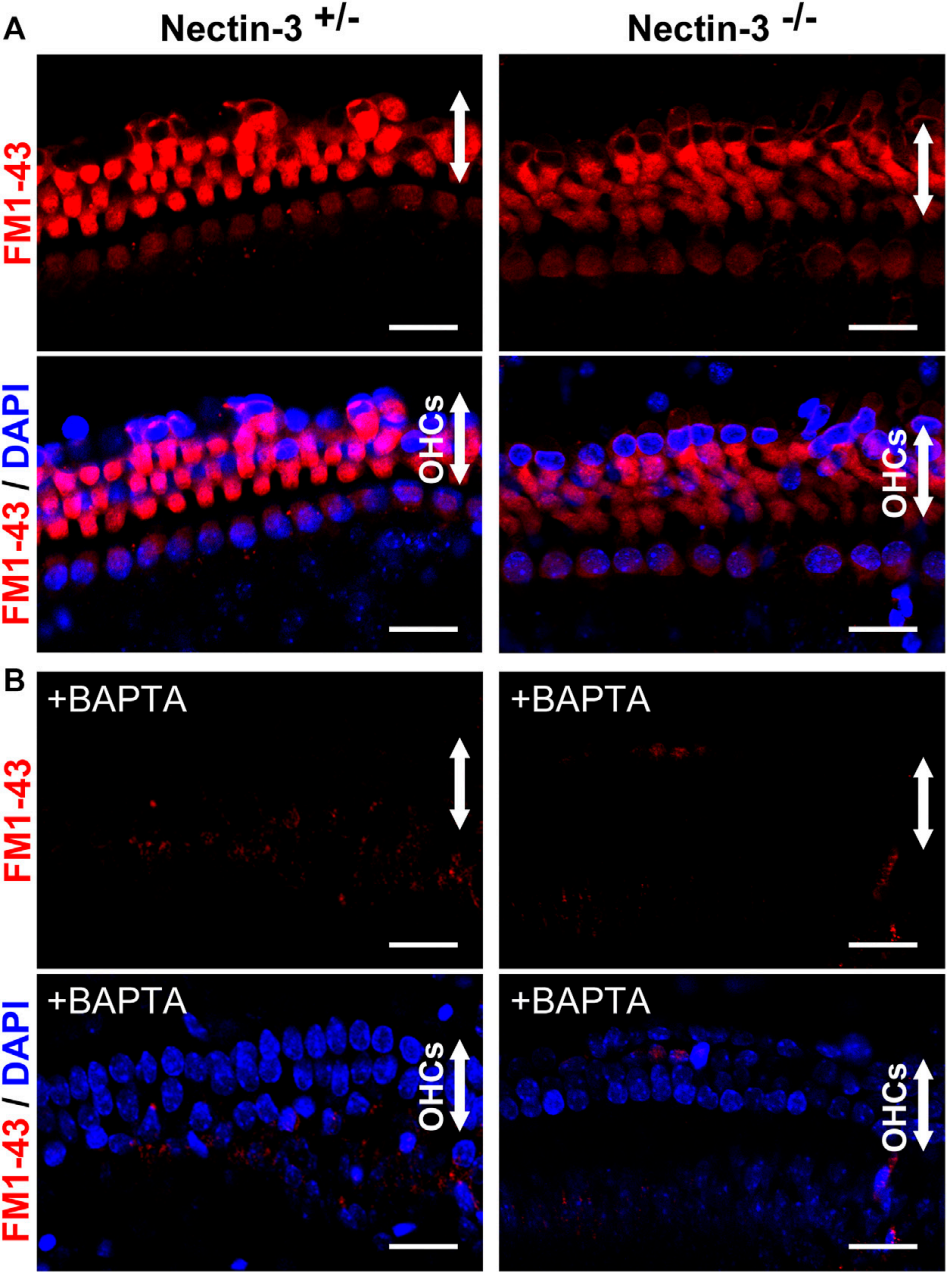
Mechanotransduction activity of neonatal nectin-3 KO mice **(A)** Fluorescence images of FM1-43 and DAPI of nectin-3^+/–^ (left) or nectin-3^−/−^ (right) mouse cochlea, were exposed to 5 μM FM1-43 for 10 s. Both nectin-3^+/–^ and nectin-3^−/−^ mouse inner and outer hair cells showed robust uptake of FM1-43. The upper panels show the fluorescent images of FM1-43, and the lower panels show the double fluorescent images of FM1-43 and DAPI. Arrows indicate the outer hair cell region of the auditory epithelia. Scale bars are 20 μm. **(B)** Fluorescence images of FM1-43 and DAPI of nectin-3^+/–^ or nectin-3^−/−^ mouse cochlea, were exposed to 5 μM FM1-43 for 10 s with the treatment of 5 mM BAPTA. Nectin-3^+/–^ and nectin-3^−/−^ mouse cochlea did not take up FM1-43. No remarkable differences were observed between nectin-3^+/–^ and nectin-3^−/−^ mice. Scale bars are 20 μm.

To evaluate the function of abnormally attached hair cells of nectin-3^−/−^ mice before degeneration, we examined the direct application of FM1-43 and its uptake in nectin-3^+/–^ and nectin-3^−/−^ cochleae on P4 (Figure 4A). Mechanotransduction activity is initiated by the opening of cation channels in hair cells, which can be visualized by the uptake of FM1-43 (Gale et al., 2001; Meyers et al., 2003; Higashi et al., 2015). We dissected the cochlea from nectin-3^+/–^ and nectin-3^−/−^ on P4 and compared FM1-43 uptake between nectin-3^+/–^ and nectin-3^−/−^ cochlear explants in the bath application. As shown in Figure 4, all cochlear hair cells were labeled throughout their cell bodies, except for the nucleus in both nectin-3^+/–^ and nectin-3^−/−^ cochleae. FM1-43 uptake into outer and inner hair cells was not affected in nectin-3^−/−^ cochlea, indicating that abnormally attached hair cells in nectin-3^−/−^ mice have functional mechanotransduction activity at least until P4. Mechanotransduction activity of hair cells is required for Ca^2+^ influx. We loaded cochlear explants with BAPTA, which inhibits Ca^2+^ influx, and confirmed that the application of BAPTA completely inhibited FM1-43 uptake in hair cells of nectin-3^+/–^ and nectin-3^−/−^ cochleae (Figure 4B). These results suggest that degeneration, rather than malfunction of hair cells, is the primary cause of hearing impairments in nectin-3^−/−^ mice.

### Apoptotic outer hair cell death in nectin-3 KO cochlea

**FIGURE 5:**
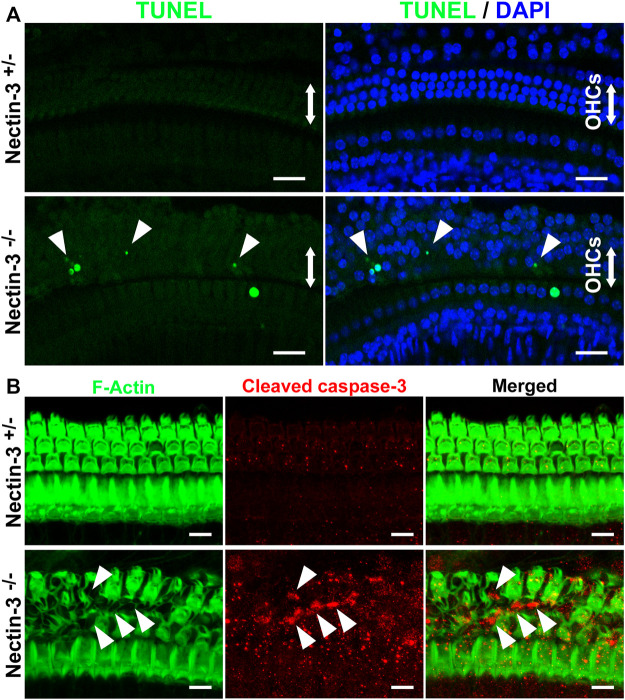
Apoptotic cell death of hair cells in nectin-3 KO mice **(A)** TUNEL assays in the middle turn of nectin-3^+/–^ and nectin-3^−/−^ cochlea on P28. Nuclei were counterstained with DAPI (blue). Note the TUNEL-positive (green) condensed nuclei in nectin-3^−/−^ cochlea. Arrowheads indicate TUNEL-positive outer hair cells. Scale bars are 20 μm. **(B)** Immunofluorescence images of the organ of Corti in the middle turn in nectin-3^+/–^ and nectin-3^−/−^ mice on P24 using an anti-cleaved caspase-3 antibody (red) and phalloidin (green). Cleaved caspase-3 signal, which indicates the induction of apoptosis, was not detected in the auditory epithelium in nectin-3^+/–^ mice. The cleaved caspase-3 signal was detected only in the outer hair cell region in nectin-3^−/−^mice. The difference in the number of TUNEL-positive and caspase-positive cells might reflect the difference in the time required to induce cell death and the time it takes for the dead cells to be removed. Arrowheads indicate the positive cells. Scale bars are 20 μm.

The number of hair cells gradually decreased in the nectin-3^−/−^ cochlea from P18 onwards (Figure 3C). To determine whether apoptosis is involved in the degeneration of hair cells in the nectin-3^−/−^ cochlea, we performed terminal deoxynucleotidyl transferase dUTP nick end labeling (TUNEL) assays on P28, when the loss of outer hair cells was expected to progress. On P28, a positive TUNEL signal was barely detected throughout the cochlea in nectin-3^+/–^ mice. In contrast, TUNEL signals were observed in the outer hair cell region of nectin-3^−/−^ mice. TUNEL-positive cells with condensed nuclei were frequently observed in the outer hair cell regions of the nectin-3^−/−^ cochlea, but not in the nectin-3^+/–^ cochlea (Figure 5A). Dissected cochleae were labeled with DAPI to identify the positions of hair cells. The number of TUNEL-positive cells was counted on P28, and the average number of TUNEL-positive cells per field was significantly larger in nectin-3^−/−^ (5.25 ± 1.26, *n* = 4) than in nectin-3^+/–^ (0.25 ± 0.5, *n* = 4, *p* < 0.001). On P9, the organ of Corti in both nectin-3^+/–^ and nectin-3^−/−^ mice had no TUNEL signals (data not shown). We also immunostained the auditory epithelium of the nectin-3^+/–^ and nectin-3^−/−^ cochleae with cleaved caspase-3 on P24. Cleaved caspase-3 is a marker of apoptosis. Cleaved caspase-3 positive cells were detected in nectin-3^−/−^ cochlea; these signals were adjacent to each other in the auditory epithelium (Figure 5B). In contrast, cleaved caspase-3 was not detected in the nectin-3^+/–^ cochlea. These results indicate that the degeneration of sensory hair cells in the nectin-3^−/−^ cochlea is caused by apoptosis.

### Disappearance of ZO proteins at the junctions between hair cells

**FIGURE 6:**
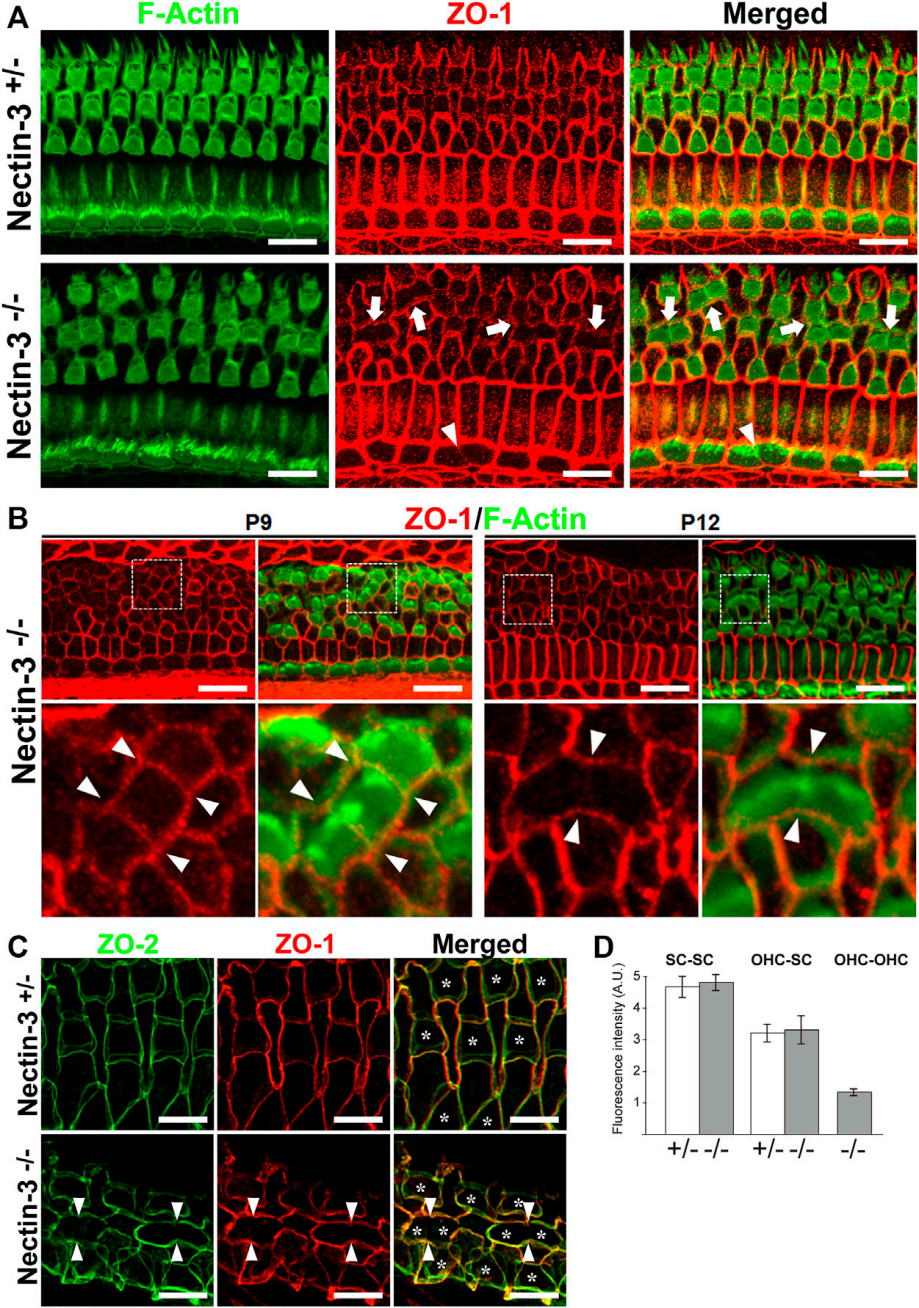
Localization of ZO proteins in the auditory epithelium in nectin-3 KO mice **(A)** Immunofluorescence images of the organ of Corti in the middle turn in nectin-3^+/–^ and nectin-3^−/−^ mice on P12 using an anti-ZO-1 antibody (red) and phalloidin (green). White arrows indicate the homotypic interfaces between attached outer hair cells, and white arrowheads indicate the homotypic interface between attached inner hair cells. Scale bars are 10 μm. **(B)** Immunofluorescence images of the organ of Corti in the middle turn in nectin-3^+/–^ and nectin-3^−/−^ mice on P9 (left) and P12 (right). (Upper) Double immunostaining for ZO-1 (red) and F-actin (green). (lower) Close-up views of the white boxed region of the upper images. White arrowheads indicate the homotypic interfaces between attached outer hair cells. Scale bars are 10 μm. **(C)** Immunofluorescence images of the organ of Corti in the middle turn in nectin-3^+/–^ and nectin-3^−/−^ mice on P21. Double immunostaining for ZO-2 (green) and ZO-1 (red). White arrowheads indicate the homotypic interfaces between attached outer hair cells and asterisks indicate the outer hair cells. Scale bars are 3 μm. **(D)** The fluorescence intensities of ZO-1 at the various junctions in the organ of Corti in nectin-3^+/–^ and nectin-3^−/−^ mice on P12 were quantified. SC-SC, the junctions between supporting cells, OHC-SC, the junctions between outer hair and supporting cells; OHC-OHC, the junctions between outer hair cells. Error bars indicate SD. Note that the junctions between outer hair cells are only detected in nectin-3^−/−^ mice.

To elucidate the mechanism of attached hair cell death in nectin-3^−/−^ mice, we hypothesized that there might be some abnormalities in the homotypic junctions between the hair cells. We examined the localization of ZO-1 and F-actin in the nectin-3^+/–^ and nectin-3^−/−^ cochleae on P12 (Figure 6A). ZO-1 is prominently localized at the homotypic junctions between supporting cells and weakly detected at the heterotypic junctions between hair and supporting cells in the auditory epithelium of nectin-3^+/–^ mice. However, in nectin-3^−/−^ mice, we found that ZO-1 disappeared from the homotypic junctions between the outer hair cells; however, F-actin was detected at the junctions (Figure 6A, arrows). ZO-1 also decreased at the homotypic junctions between inner hair cells (Figure 6A, arrowheads). The signal intensity of ZO-1 at the junctions between the supporting cells and between the supporting and outer hair cells did not change (Figure 6D). These observations indicated that ZO-1 specifically decreased at the homotypic junctions between the outer and inner hair cells on P12. We also examined the localization of ZO-1 from P9 to P12 and found that ZO-1 was detected on P9 and disappeared from the homotypic junctions between the hair cells on P12 in nectin-3^−/−^ mice (Figure 6B). ZO proteins are essential for TJ (Tight junction) formation in epithelial cells (Umeda et al., 2006), and proper TJ formation is required to maintain ion homeostasis around cochlear hair cells and ensure their survival (Ben-Yosef et al., 2003; Nakano et al., 2009). As ZO-1/2 play an essential role in the formation of TJs, we examined the localization of ZO-2 in the nectin-3^+/–^ and nectin-3^−/−^ cochleae on P21 (Figure 6C). ZO-2 was distributed at the boundaries between hair and supporting cells, as well as between adjacent supporting cells. ZO-2 was distributed from apical to lateral contacts between outer hair and supporting cells in a double-lined fashion in the nectin-3^+/–^ cochlea on P21. However, in nectin-3^−/−^ mice, we found that ZO-2 also disappeared from the homotypic junctions between the outer hair cells on P21 (Figure 6C, arrowheads). Based on these observations, we hypothesized that aberrant attachment of outer hair cells causes abnormal TJ formation.

### Impairment of tight junction formation at the junctions between hair cells

**FIGURE 7:**
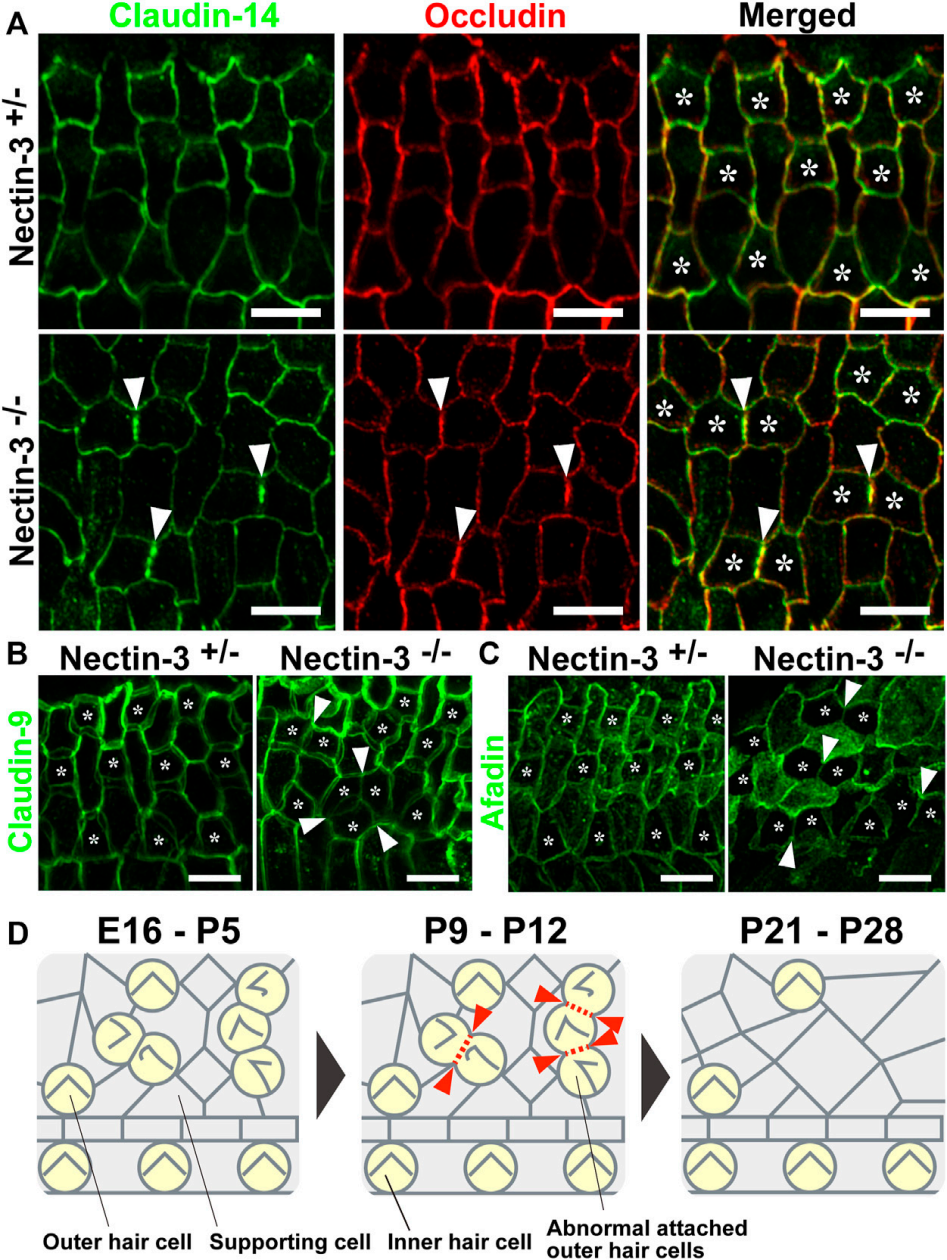
Localization of claudin-14, occludin, claudin-9, and afadin in the auditory epithelium in nectin-3 KO mice **(A)** Immunofluorescence images of the organ of Corti in the middle turn in nectin-3^+/–^ and nectin-3^−/−^ mice on P21 using an anti-claudin-14 antibody (green) and anti-occludin (red). **(B)** Immunofluorescence images of the organ of Corti in the middle turn in nectin-3^+/–^ and nectin-3^−/−^ mice on P21 using an anti-claudin-9 antibody. **(C)** Immunofluorescence images of the organ of Corti in the middle turn in nectin-3^+/–^ and nectin-3^−/−^ mice on P21 using an anti-afadin antibody. White arrowheads indicate the homotypic interfaces between attached outer hair cells, and asterisks indicate the outer hair cells. Scale bars are 5 μm. **(D)** Schematic illustrations of the cellular patterns and TJ loss in the auditory epithelium of nectin-3 KO mice. Red arrowheads indicate the positions of homotypic junctions between outer hair cells. Red dotted lines indicate the loss of TJ components from the homotypic junctions between hair cells. See also the Discussion for details.

In the auditory epithelium, the junctions between the outer hair and supporting cells and the canonical TJ and AJ (Adherens junction) proteins are recruited to a single junction and form a hybrid tight junction with an adherens junction, which is called a tight-adherens junction (Nunes et al., 2006). In wild-type mice, TJ components such as occludin and claudin-9 and -14 are localized at the junctions between hair and supporting cells (Kitajiri et al., 2004; Nunes et al., 2006). TJ complex at the apex of the reticular lamina requires claudin-9 and -14 as a cation-restrictive barrier to maintain the proper ionic composition of the fluid surrounding the basolateral surface of outer hair cells (Ben-Yosef et al., 2003; Nakano et al., 2009). To examine TJ formation between the hair cells in nectin-3^−/−^ mice, we immunostained the auditory epithelium with antibodies against occludin and claudin-14 (Furuse et al., 1993; Kitajiri et al., 2004) (Figure 7A). In the nectin-3^+/–^ cochlea, occludin and claudin-14 were uniformly distributed at the boundaries between hair and supporting cells, as well as between adjacent supporting cells on P21. The signals for claudin-14 and occludin appeared as a single line at the cell boundary. However, in nectin-3^−/−^ cochlea, the signals for occludin and claudin-14 were discontinuous at the homotypic junctions between the outer hair cells, resulting in an abnormal accumulation of these molecules on P21 (Figure 7A, arrowheads). Moreover, the localization of claudin-14 was often lost between the hair cells. We also examined the localization of claudin-9 in the nectin-3^−/−^ cochlea (Figure 7B). In the nectin-3^+/–^ cochlea, claudin-9 was distributed from apical to lateral contacts between all cells in a double-lined fashion, and often visualized as tilted intercellular boundaries on P21. The distribution pattern of claudin-9 was similar to that of ZO-2. However, in the nectin-3^−/−^ cochlea, the signal for claudin-9 weakened at the homotypic junctions between the outer hair cells, and the characteristic double-lined signal decreased (Figure 7B, arrowheads). The change in the distribution pattern suggested that claudin-9 decreased from the lateral contacts between the hair cells and that the junctional structure between the hair cells was affected. Collectively, these observations suggest that TJs formed between the hair cells are disorganized in the nectin-3^−/−^ cochlea.

To understand the mechanism underlying the regulation of TJ formation between hair cells, we examined the localization of afadin in the nectin-3^+/–^ and nectin-3^−/−^ cochleae on P21 (Figure 7C). Afadin, which is the cytoplasmic binding partner of nectins, regulates TJs formation cooperatively with ZO-1 in epithelial cells (Takekuni et al., 2003; Ooshio et al., 2007). In the nectin-3^+/–^ cochlea, afadin was uniformly distributed at the boundaries between hair and supporting cells, as well as between supporting cells. Afadin was distributed from the apical to lateral contacts between all cells in a double-lined fashion on P21, and the distribution pattern was similar to that of claudin-9. However, in the nectin-3^−/−^ cochlea, the signal for afadin decreased at the homotypic junctions between the outer hair cells (Figure 7C, arrowheads). These results suggest that the decrease in TJ components from the homotypic junctions between outer hair cells was due to the decreased localization of ZO proteins and afadin from the homotypic junctions.

## Discussion

The auditory epithelium of the inner ear comprises mechanosensory hair cells and various types of supporting cells. These cells are arranged in a checkerboard-like pattern and have been evolutionarily conserved among a wide range of species. The checkerboard-like pattern of the auditory epithelium has long been thought to be necessary for auditory function but has never been directly examined. In the present study, we demonstrated that the disruption of checkerboard-like cellular patterns in the auditory epithelium causes hearing defects. In nectin-3 KO mice, the differentiation of hair and supporting cells was normal in the early developmental stage; however, many hair cells were aberrantly attached and the checkerboard-like cellular pattern was disorganized from early developmental stage. Hearing defects in nectin-3 KO mice were caused by apoptosis of attached outer hair cells. Aberrant attachment between outer hair cells induced a defect in TJ formation after the postnatal stage, and failed barrier formation causes apoptotic hair cell death (Figure 7D). The disruption of a checkerboard-like pattern in nectin-3 KO mice is observed regardless of hair cell death in the early developmental stage. However, the hair cell death might also enhance the disruption of the checkerboard-like pattern of the auditory epithelium, because the hair cell death induces a rearrangement of the cellular pattern of the auditory epithelium. These results provide the first evidence that the checkerboard-like cellular pattern of the auditory epithelium plays a role in the structural basis ensuring the survival of sensory hair cells and a robust hearing function.

Hearing loss due to failure of TJ formation has been extensively investigated in mouse models. Claudin-9 and -14 and occludin in mutant mice show rapid degeneration of cochlear hair cells, resulting in hearing loss (Ben-Yosef et al., 2003; Nakano et al., 2009; Kitajiri et al., 2014). In these KO mice, the ultrastructural changes in TJs are thought to affect the paracellular permeability of ions or small molecules, resulting in a toxic microenvironment for cochlear hair cells. The phenotypes of degeneration of cochlear hair cells in nectin-3 KO mice were different from those in mutant mice in the following points: degeneration timing of outer hair cells, degeneration of inner hair cells, and degeneration speed of hair cells. In TJ-associated molecules of these mutant mice, nearly all outer hair cells were lost by P21, and at least half of the total number of inner hair cells were lost by the same period. The outer hair cells in these mutant mice rapidly degenerate within 7 days from P10–P13 (Ben-Yosef et al., 2003). However, in nectin-3 KO mice, the outer hair cells slowly degenerated from P18, and the unattached outer and inner hair cells were retained. In nectin-3 KO mice, the homotypic adhesion between hair cells is frequently observed in outer hair cells, while the homotypic adhesion between inner hair cells is rarely observed (Togashi et al., 2011). This would be the reason why there was almost no cell death of inner hair cells in nectin-3 KO mice. These observations suggest that apoptosis of hair cells in nectin-3 KO was not simply caused by loss of TJs due to nectin deficiency, but rather by cell death caused by abnormal TJ formation induced by aberrant attachment between hair cells. The slower rate of hair cell death in the nectin-3 KO cochlea might reflect the limited number of attached hair cells. The lateral wall of cochlea (stria vascularis) also contributes to auditory function and maintenance of homeostasis in the cochlear fluid through the generation of endocochlear potential (EP) (Yu et al., 2021). The tight junction formation in the stria vascularis is important for the generation of EP (Gow et al., 2004). Although there is no report on the expression of nectin-3 in the stria vascularis, the function of other nectin in stria vascularis formation may be worth further investigation.

In nectin-3 KO cochlea, the localization of ZO proteins significantly decreased at the homotypic junctions between outer hair cells (Figure 6). ZO proteins are required for TJ formation (Umeda et al., 2006). The reduction in ZO proteins induced a decrease in TJ components at the homotypic junctions between outer hair cells (Figures 7A,B). However, TJ components such as claudins and occludin are not immediately lost and remain at the homotypic junctions between the hair cells until around P21. The reason for the time lag between the disappearance of the ZO molecules and of the TJ molecules is unknown. Previous cell biological studies suggested that nectin and afadin cooperatively recruit ZO-1, claudin, and occludin to the apical side of AJs, resulting in the formation of TJs (Ooshio et al., 2010). Our previous study showed that nectin-1 and -2 and afadin were concentrated at the boundaries between hair cells in the nectin-3 KO cochlea on P1 (Togashi et al., 2011; Fukuda et al., 2014). However, the expression of nectin-1 in the outer hair cells gradually decreased after birth, which was accompanied by a concomitant decrease in afadin between outer hair cells (Figure 7C). We could not detect any signal for nectin-1 in the apical junctions in wild-type and nectin-3 KO cochlea on P12. Thus, decreased expression of nectin-1 in hair cells may be due to a cell-intrinsic mechanism.

Mutations in nectin-1 gene cause cleft lip/palate ectodermal dysplasia, Margarita Island ectodermal dysplasia, and Zlotogora–Ogür syndrome, which is characterized by cleft lip/palate, syndactyly, intellectual disability, and ectodermal dysplasia (Suzuki et al., 2000; Sozen et al., 2001). It has been reported that patients with these disorders have mild, non-congenital, deafness (Bustos et al., 1991). In nectin-1 KO mice, similar patterning defects were observed in the auditory epithelium as in nectin-3 KO mice, but the effects in nectin-1 KO were much milder than those in nectin-3 KO mice (Togashi et al., 2011). Mild hearing impairment in patients with cleft lip/palate ectodermal dysplasia, Margarita Island ectodermal dysplasia, and Zlotogora–Ogür syndrome might reflect fewer abnormal attachments between hair cells.

At the apical surface of the auditory epithelium, sensory hair and supporting cells are interconnected by tight and adherens junctions to form the reticula lamina (Gulley and Reese, 1976; Nunes et al., 2006). Individual supporting cells contain specialized arrays of cytoskeletal filaments known as microtubule stalks, which provide rigid support to hair cells (Slepecky et al., 1995). The tight seal of hair and supporting cells at the reticular lamina is essential for structural integrity of the organ of Corti. The mosaic patterns might provide a structural basis for hair cell mechanotransduction. The basic structures and functions of the sensory epithelia of the ear, nose, and eye are highly conserved in evolution, not only from one vertebrate to another, but also between vertebrates and invertebrates. Our study provides the foundation for future research on understanding how the structural basis provided by the mosaic cellular pattern in sensory epithelium play a role in physiological functions.

## Materials and methods

### Mice

C57BL/6 mice were purchased from CLEA Japan Inc. Nectin-3 KO mice were generated by homologous recombination using targeting vectors designed to delete the exon 1 of the nectin-3 gene (Inagaki et al., 2005). All the experimental procedures were approved and performed according to the Kobe University Animal Experimentation Regulations (Permission numbers: P140108 and P180710).

### Auditory brainstem response and distortion product otoacoustic emission measurements

Auditory brainstem response (ABR) measurements were performed according to the method described previously (Fujita et al., 2012). Mice were anesthetized by injecting a mixture of medetomidine (37.5 μg/kg), midazolam (10 mg/kg), and butorphanol (0.5 mg/kg). ABR measurement was performed using waveform storing and stimulus control with Scope software on the PowerLab system (PowerLab2/26; AD Instruments), and electroencephalogram (EEG) recording was performed with the extracellular amplifier AC PreAmplifier (P-55; Astro-Med). Sound stimuli were produced by a coupler-type speaker (ES1spc; Bio Research Center) inserted into the external auditory canal of mice. Tone burst stimuli, with a 0.2 m rise/fall time (cosine gate) and 1-ms flat segment at frequencies of 4, 8, 16, and 32 kHz, were generated, and the amplitude was specified by a sound generator and attenuation Real-Time Processor and Programmable Attenuator (RP2.1and PA5; Tucker-Davis Technologies). Sound-level calibrations were performed using a Sound Level Meter (NA-42; Rion). For recording, stainless steel needle electrodes were placed at the vertex and ventrolateral to the left and right ears.

DPOAEs were measured by commercial instrumentation ER-10X System (Etymotic Research) combined with custom software EMAV (Neely and Liu, 2013). DPOAEs were tested according to the primary tones, f1, and f2, which were set at an f2/f1 ratio of 1.2, with sound pressure levels of 65- and 55-dB SPL respectively. A probe was inserted into the ear canal and DPOAE amplitude was measured at f2 frequencies of 12, 16, 24, and 32 kHz and plotted after subtracting baseline noise.

### Immunofluorescence microscopy

To prepare whole-mount samples of the cochlea from mice on P9-P28 and 1 year after birth, the cochlea was dissected out and fixed with 4% paraformaldehyde (PFA) in Hank’s balanced salt solution containing 1 mM Ca^2+^ and Mg^2+^, containing 10 mM HEPES buffer (HBSS CM+) at room temperature for 30 min or 10% TCA in water at 4°C for 10 min. After fixation, the samples were blocked with a blocking solution (0.25% Triton X-100 and 5% normal goat serum in Tris-buffered saline (TBS)) at 4°C overnight, followed by incubation with primary antibodies (Abs) in 5% goat serum or in “Can Get Signal” immunoreaction enhancer solution (TOYOBO) at room temperature for 2 h. The samples were then incubated in secondary Abs in 5% goat serum at room temperature for 75 min. These samples were flat mounted on a slide glass with glycerol gelatin (Sigma-Aldrich).

The antibodies used were rabbit anti-cleaved-Caspase-3 (Asp175) pAb (Cell Signaling Technology), rabbit anti-ZO-1 pAb (Invitrogen), rat anti-ZO-1 mAb (clone R40.76, Santa Cruz Biotechnology), rabbit anti-ZO-2 pAb (Invitrogen), rabbit anti-afadin pAb (Sigma-Aldrich), rat anti-occludin mAb (clone MOC37, generous gift from Dr. Furuse) (Furuse et al., 1993), rabbit anti-claudin-14 pAb (a generous gift from Dr. Furuse) (Kitajiri et al., 2004), rabbit anti-claudin-9 pAb (a generous gift from Dr. Furuse) (Kitajiri et al., 2004). Primary Abs have been visualized with goat fluorochrome-conjugated secondary Abs. The fluorochromes used were Alexa Fluor 488, 555, and 647 (Thermo Fischer), and Cy3 (Merck Millipore). F-Actin was visualized by use of Alexa 488-conjugated phalloidin (Thermo Fischer). Images were obtained with a confocal microscope (LSM700 and LSM900; Carl Zeiss Microscopy) equipped with a 40× NA 1.3, a 40× NA 1.2, and a 20× NA 0.8 lens using ZEN software (Carl Zeiss Microscopy), and these images were analyzed with the same software and processed with Adobe Photoshop.

### FM1-43 uptake analysis

Cochleae were dissected from P4 nectin-3^+/–^ and nectin-3^−/−^ mice in HBSS CM+ and explanted onto a glass-bottom dish (Matsunami Glass) previously coated with Cell-Tak (Corning). Explants were incubated in DMEM/F12 at 37°C with 5% CO_2_ for 1 h until they were firmly attached to the culture dish. FM1-43 (Thermo Fischer) was applied to the mounted tissue according to the method previously described with some modifications (Kawashima et al., 2011; Higashi et al., 2015). Explants were washed three times with HBSS CM+, then 5 μM FM1-43 in HBSS CM+ was applied for 10 s at room temperature, followed immediately by 4-time washes (within 1 min) with HBSS CM+. When testing the effects of BAPTA, HBSS CM+ was replaced with HBSS containing 5 mM BAPTA in every step. FM1-43 fluorescence images from middle turns of cochleae were captured 5 min after FM1-43 treatment.

### Transferase dUTP nick end labeling assay

Apoptotic cells were detected using the TUNEL method with an Apoptag Fluorescein *In Situ* Apoptosis Detection Kit (Thermo Fischer). After fixation using 4% PFA, samples were permeabilized with 0.5% Triton X-100 in HBSS CM + for 30 min, processed using the supplier’s TUNEL method, and then incubated with DAPI for 10 min.

### Statistical analysis

All data are presented as the mean ± standard deviation. Statistical analysis of two groups was performed using a two-tailed unpaired Student t-test. When the variance between samples was unequal, Welch’s test was used. A *p*-value of <0.05 was considered statistically significant.

## Data Availability

The raw data supporting the conclusion of this article will be made available by the authors, without undue reservation.

## Supplementary material

SUPPLEMENTARY FIGURE S1 Morphology of outer hair cells in nectin-3 KO mice at P15. Close-up views of the stereocilia, visualized by phalloidin staining, in attached outer hair cells on P15. (Left) nectin-3^+/–^, (right) nectin-3^–/–^. Scale bars are 5 μm.

## Abbreviations

AJ, adherens junction; E, embryonic day; P, postnatal day; TJ, tight junction.
