# Supplementary figures and images for: Hearing loss in mice with disruption of auditory epithelial patterning in the cochlea
Source: Front Cell Dev Biol. 2022 Dec 8;10:1073830. doi: 10.3389/fcell.2022.1073830 (PMC9773838; doi:10.3389/fcell.2022.1073830)

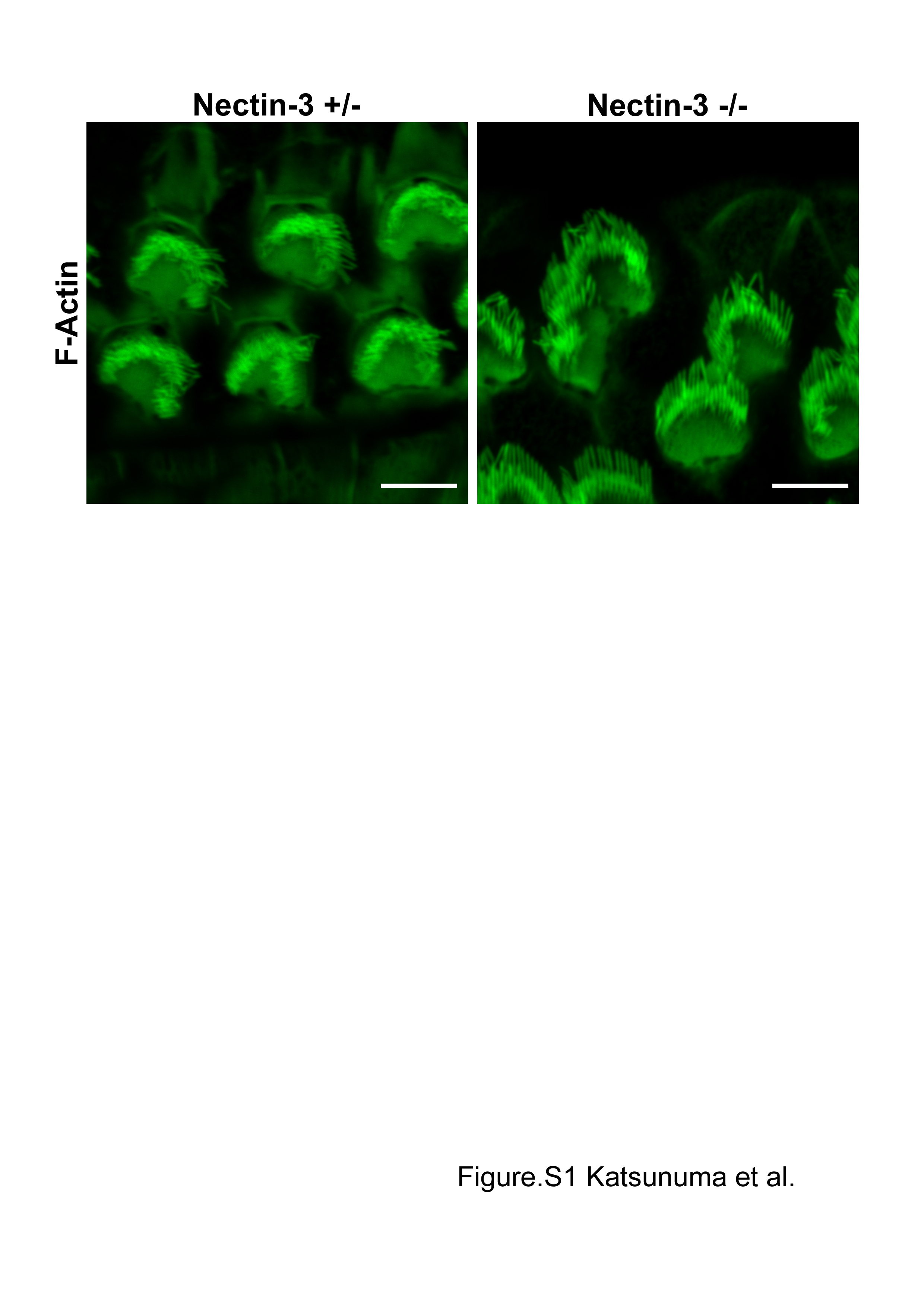

Supplement: Supplementary file 1 [file Image1.TIF]
